# Internal mammary node irradiation in early breast cancer – target coverage and implications on dose to organs at risk

**DOI:** 10.2340/1651-226X.2025.43716

**Published:** 2025-07-30

**Authors:** Lovisa Berg, Jeanette Sporre, Elisabeth Kjellén, Sofie Ceberg, Elinore Wieslander, Sara Alkner

**Affiliations:** a Radiation Physics, Department of Hematology, Oncology and Radiation Physics, Skåne University Hospital, Lund, Sweden; b Department of Oncology, Institute of Clinical Sciences, Faculty of Medicine, Lund University, Lund, Sweden; c Clinic of Oncology, Department of Hematology, Oncology and Radiation Physics, Skåne University Hospital, Lund, Sweden; d Medical Radiation Physics, Department of Clinical Sciences Lund, Lund University, Lund, Sweden

**Keywords:** Breast cancer, adjuvant radiotherapy, internal mammary nodes, treatment planning

## Abstract

**Purpose:**

Indications for radiotherapy (RT) of the internal mammary nodes (IMN) in early breast cancer vary between countries. While studies indicate benefits, IMN RT increases the dose to the heart and lungs, and the risk-benefit ratio of this treatment is debated. This study investigates how IMN RT affects dose to organs at risk (OAR) and pneumonitis incidence in a clinical setting.

**Methods:**

This retrospective study includes breast cancer patients receiving adjuvant locoregional RT with and without IMN included in the target volume at Skåne University Hospital, Sweden, from 2018 to 2021. Treatment plans followed national dose-volume criteria, prioritizing lung and heart over IMN coverage. A total of 247 treatment plans for locoregional RT with IMN were compared to 397 without. Dose to OAR, IMN coverage and pneumonitis incidence were investigated.

**Results:**

The mean ipsilateral lung dose increased by 2.7 Gy with IMN RT (*p* < 0.001), and the mean heart dose (left-sided treatment) by 0.5 Gy (*p* < 0.001). Both irradiated and treated volume in relation to planning target volume (PTV) increased with ~20% (*p* < 0.001). Desired IMN coverage was achieved in 76% of the plans, with lung dose exceeding recommended constraints as the primary reason for decreased target coverage in the remaining plans. Of the 220 patients with follow-up of ≥6 months, 2 (0.9%) were diagnosed with pneumonitis grade 2.

**Interpretation:**

Introduction of IMN RT primarily resulted in an increased lung dose. However, rate of symptomatic pneumonitis was low. Most patients achieved desired IMN coverage using 3D-CRT, with lung dose being the limiting factor.

## Introduction

Breast cancer (BC) is the most common cancer among Swedish women, constituting 26% of all female cancer diagnoses. Over the past two decades, the number of cases has consistently increased by 2% annually [1]. To improve prognosis in early-stage BC, patients undergo postoperative locoregional radiotherapy (RT). RT is a research field in constant development, and a newly published meta-analysis shows that locoregional RT significantly reduced BC mortality and all-cause mortality in trials done after the 1980s, but not in older trials [2]. These results indicate that RT has become a more tailored and effective treatment during the last decades.

However, although RT improves survival on a group level the effect for the individual is uncertain and the treatment may cause side effects. Adding RT increases arm morbidity, lymphedema and impairs cosmetic outcome after breast reconstruction. RT may also result in both acute (radiation pneumonitis and skin problems) and late side effects (heart disease, secondary malignancies, and hypothyroidism) [3–8]. In addition, modern systemic therapy has improved prognosis and locoregional recurrences are today rare events [9]. This has led to a debate regarding adjuvant locoregional RT treatment guidelines, encompassing patient selection and inclusion of specific nodal levels in the treatment volume based on patient and tumor characteristics. The central question remains: How do we optimize adjuvant locoregional RT to provide a tailored and individualized treatment for each patient, balancing the benefits of cancer control while minimizing side effects. Currently, several ongoing studies are investigating this question to develop the next generation of treatment strategies [10–13].

One example of variation in treatment guidelines between countries and sites is the inclusion of the internal mammary nodes (IMN) in the RT target volume [14, 15]. The risk of metastasis in the IMN is increased not only if there are metastasis in the axillary lymph nodes but also with tumor size and a medial location of the tumor [16]. Studies indicate that, even with modern systemic therapy, IMN RT remains important for patients with a high risk of recurrence [17, 18]. In addition, IMN RT was the main comparator in a recent meta-analysis showing a benefit of increased regional lymph node target volumes [2]. On the other hand, adding IMN to the RT field increases dose to organs at risk (OAR), such as the heart and ipsilateral lung.

In 2018 the Swedish national BC guidelines for RT were revised to include IMN in the RT target volume for patients with ≥4 lymph node metastases, or 1–3 lymph node metastases and a central/medial tumor location in the breast. A notable addition to the guidelines was to prioritize heart and ipsilateral lung constraints over IMN coverage [19, 20].

The aim of this study is to evaluate, through retrospective analysis of patients receiving locoregional BC-RT at Skåne University Hospital, how the inclusion of the IMN in the target volume affects dose to OAR and normal tissue, and the incidence of pneumonitis in the clinical setting. Previous studies on this matter are sparse, and it will give us valuable information for evaluation of risk-benefits with IMN RT. We also investigate the ability to meet the clinical criteria for IMN coverage without surpassing the national dose limits for the heart and ipsilateral lung.

## Materials and methods

**Figure 1:**
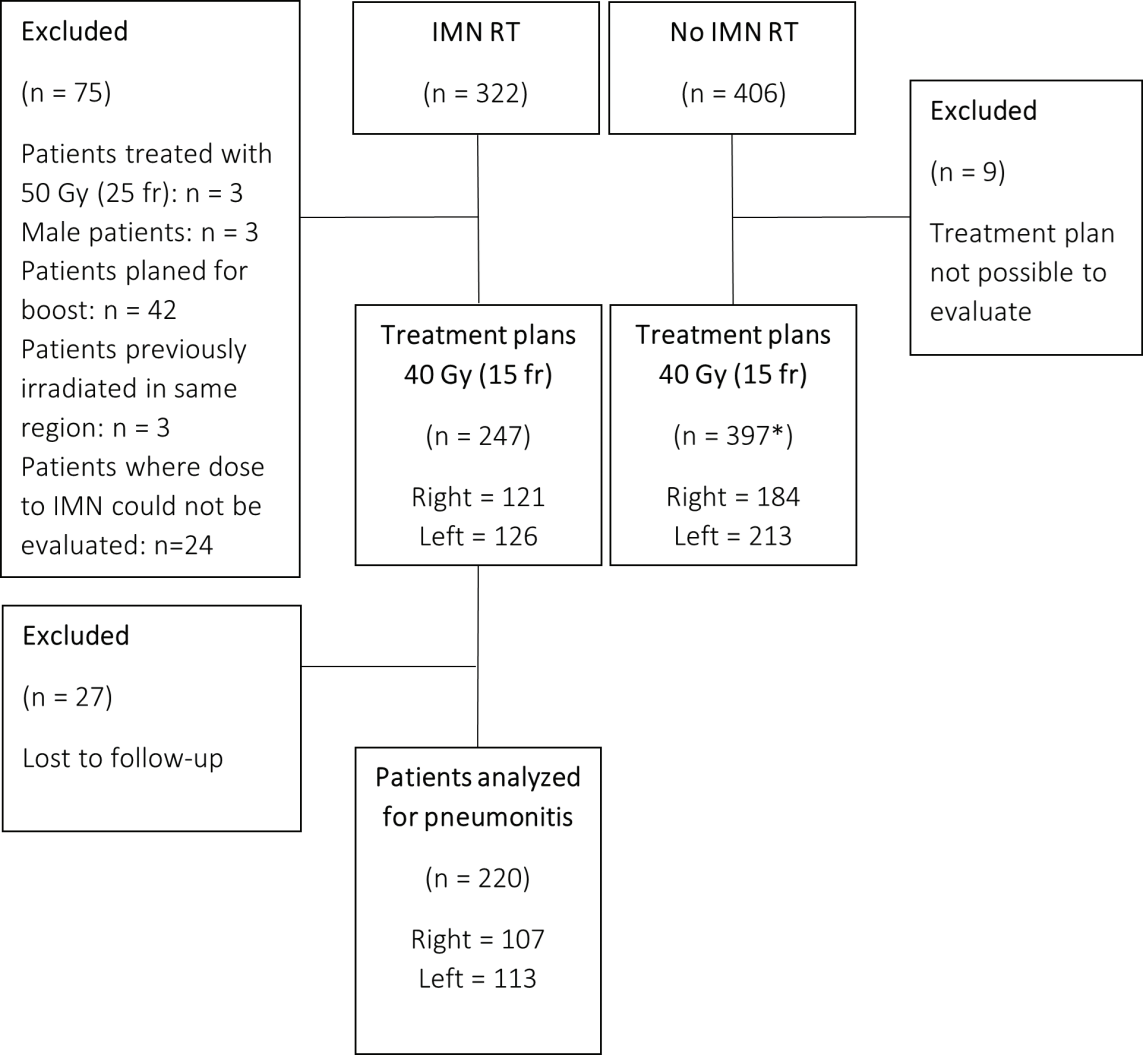
Consort diagram for patients with early-stage breast cancer registered for locoregional radiotherapy with and without internal mammary node irradiation. *Two bilateral patients, which results in 395 patients and 397 treatment plans. fr: fraction (fr); IMN: internal mammary nodes; *n*: number; RT: radiotherapy.

This retrospective cohort study is reported in accordance with the STROBE (Strengthening the Reporting of Observational Studies in Epidemiology) guidelines. It includes all female patients with early-stage BC receiving adjuvant locoregional RT (breast/chest wall and regional lymph nodes) with and without the IMN included in the target volume at Skåne University Hospital, from 27th of Aug 2018 to 19th of Oct 2021. The number of patients in each group and exclusion criteria are presented in Figure 1. The study has been approved by the Swedish Ethics Review Authority (LU2013/742 and 2023-02667-01).

All patients underwent a computed tomography (CT) scan with a slice thickness of 3 mm and arms fixated above their heads, in accordance with clinical standards. Patients scheduled for IMN RT were offered gated treatment with deep inspiration breath hold (DIBH) independent of treatment side, in order to spare dose to OAR. For patients without IMN RT, DIBH was used only for left-sided treatments and for bilateral patients. The patient´s breathing motion and reference surface in DIBH were obtained during the CT scan with the Sentinel^TM^ system (C-rad Positioning AB, Uppsala, Sweden), while gating during treatment was performed using the optical surface scanning system Catalyst^TM^ (C-rad Positioning AB, Uppsala, Sweden).

The target volumes were defined according to the guidelines of the Swedish Breast Radiotherapy Group (SBRG) and the European Society of Radiation Oncology (ESTRO) [20, 21]. Treatment plans were created, using a 3D-conformal radiotherapy (3D-CRT) technique. In the cranial section of the target, which includes the axilla and supraclavicular region, the fields were in the anteroposterior orientation. In the caudal section, covering the breast or chest wall, a tangential field setting was used. The treatment planning system used was Eclipse (version 13.6 and 15.6, Varian Medical Systems, Palo Alto, CA, USA) and the anisotropic analytical algorithm. The prescribed dose was 40.05 Gy in 15 fractions, 5 days a week.

Each treatment plan aimed to fulfill the dose constraints defined by SBRG. The desired coverage for the planning target volume (PTV), including the axilla, supraclavicular region, and breast or chest wall, was D_98%_ ≥ 93%. The PTV margin for the clinical target volume of the IMN (CTV_IMN_) was 5 mm, and the PTV structure was used during treatment planning. However, the evaluation and dose constraints were based on CTV_IMN_, where the goal was to achieve V_90%_ ≥ 98%, while also prioritizing dose constraints to the heart and the ipsilateral lung [20]. To fulfill dose constraint to heart and lung, it was thus allowed to decrease target coverage to CTV_IMN_. This was done by reducing dose coverage in the most caudal border of the structure, moving upward until OAR dose constraints were met. Furthermore, full dose was not pursued in the uppermost cranial part of the IMN, situated behind the sternoclavicular joint [21].

Mean doses (D_mean_), volume criteria (V_xGy_), as well as complete cumulative dose-volume histograms (DVH) were derived for the ipsilateral lung and the heart using an in-house developed software. For PTV, near minimum doses (D_98%_) were extracted. Other examined dose-volume parameters were Treated Volume (TV) and Irradiated Volume (IV), defined by ICRU Report No. 50 [22]. The TV is the volume enclosed by an isodose selected and specified by a radiation oncologist as being appropriate to achieve the purpose of treatment, in our case the 90% isodose line. The IV is the volume that receives a dose that is considered significant in relation to normal tissue tolerance, in our case 50% of prescribed dose or more. To account for PTV variation and enable comparison between patients, the IV and TV were divided by the PTV volume to create a factor that quantifies the relationship between these parameters in terms of size.

A subset of 100 randomly selected individuals out of the cohort underwent a more detailed investigation focusing on OARs that had not been delineated in clinical practice. This group included 50 patients with IMN RT and 50 without. Within each subgroup, 25 patients received left-sided treatment, and 25 right-sided treatment. Additional OAR delineated included the thyroid, humeral head, contralateral lung, contralateral breast, and the entire esophagus.

Comparison between groups were performed with independent sample t-test (two-sided), or when applicable the non-parametrical Mann–Whitney test. Significance level was set at *p* = 0.05. The statistical program used was IBM SPSS Statistics (version 29.0, IBM Corp. Armonk, NY, USA).

Patient characteristics and the frequency of symptomatic pneumonitis (grade 2 or higher) in the group with IMN RT were identified by review of the patients’ medical records. Pneumonitis was defined according to the Common Terminology Criteria for Adverse Events version 5.0 (CTCAE v 5.0) [23]. Radiology after treatment was not routinely performed. Only patients with a follow-up of at least 6 months after completing radiation were included in the analysis of pneumonitis.

## Results

### Organs at risk

**Figure 2:**
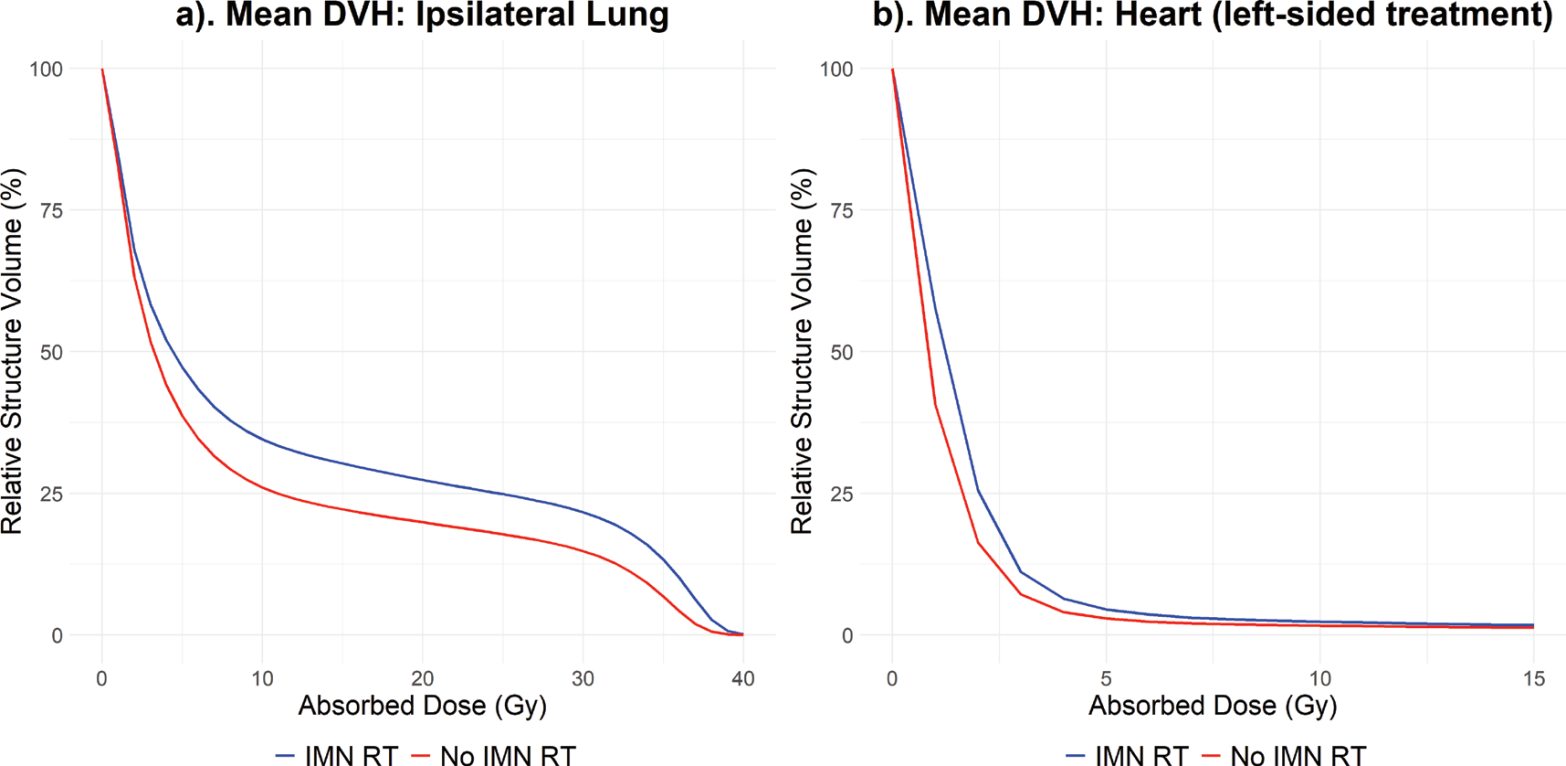
Mean DVH for the ipsilateral lung (a) and the heart (left-sided) (b) for patients with and without internal mammary node irradiation. DVH: dose-volume histogram; Gy: gray; IMN: internal mammary nodes; RT: radiotherapy.

The results for DVH metrics for heart and ipsilateral lung are presented in Table 1. Mean DVH for mentioned structures are shown in Figure 2.

**Table 1:** Dose-volume data for the lung, heart, IV, and TV.

	IMN RT (n = 247) Mean (95% CI)	No IMN RT (n = 397) Mean (95% CI)	*p* *
**D_mean, Lung_ [Gy]**			
Ipsilateral side	12.0 (11.8–12.3)	9.3 (9.2–9.5)	< 0.001
Left-side treatment	12.5 (12.2–12.7)	9.2 (8.9–9.4)	< 0.001
Lung sin	*n* = 126	*n* = 213	
Right-side treatment	11.6 (11.3–12.0)	9.5 (9.3–9.8)	< 0.001
Lung dx	*n* = 121	*n* = 184	
**V_16Gy, Lung_ [%]**			
Ipsilateral side	29.6 (29.0–30.3)	21.7 (21.1–22.2)	< 0.001
Left-side treatment	31.1 (30.3–31.8)	21.3 (20.6–22.1)	< 0.001
Lung sin	*n* = 126	*n* = 213	
Right-side treatment	28.1 (27.1–29.2)	22.1 (21.2–22.9)	< 0.001
Lung dx	*n* = 121	*n* = 184	
**D_mean, Heart_ [Gy]**			
Left-side treatment	2.0 (1.8–2.2)	1.5 (1.4–1.6)	< 0.001
	*n* = 126	*n* = 213	
Right-side treatment	0.8 (0.7–0.8)	0.5 (0.5–0.5)	< 0.001
	*n* = 109	*n* = 103	
**V_17Gy, Heart_ [%]**			
Left-side treatment	1.5 (1.1–1.9)	1.2 (0.9–1.4)	0.1
	*n* = 126	*n* = 213	
Right-side treatment	0.0 (0.0–0.1)	0.0 (0.0–0.0)	0.1
	*n* = 109	*n* = 103	
**IV[cc]**	3103 (3002–3204)	2783 (2706 – 2860)	< 0.001
**IV [cc]/PTV [cc]**	4.0 (3.9 – 4.1)	3.3 (3.2– 3.4)	< 0.001
**TV[cc]**	2059 (1977 – 2141)	1823 (1759 – 1886)	< 0.001
**TV [cc]/PTV [cc]**	2.6 (2.5 – 2.7)	2.1 (2.1 – 2.1)	< 0.001

* *P*-value for an independent sample *t*-test (two-sided) was calculated for all parameters except V_17Gy,Heart_, where a non-parametrical Mann–Whitney test was used.

CI: confidence interval; cc: cubic centimeter; Gy: gray; IMN: internal mammary nodes; IV: irradiated volume; RT: radiotherapy; TV: treated volume.

For the ipsilateral lung, both D_mean_ and V_16Gy_ were significantly (*p* < 0.001) higher in patients receiving IMN RT compared to patient not receiving IMN RT, with a difference in mean value of 2.7 Gy and 7.9%, respectively. D_mean_ and V_16Gy_ difference were more pronounced for left-side treatments, resulting in a difference in mean value of 3.3 Gy and 9.8%, respectively, for treatments with versus without IMN RT. For right-side treatment, the difference in mean value was 2.1 Gy and 6.0%.

A significant dose difference for the heart was seen for both treatment sides (*p* < 0.001). IMN RT increased the mean of D_mean_ with 0.5 Gy for left-sided treatment and 0.3 Gy for right-sided treatment. There was no significant difference for V_17Gy_.

Doses to additional OAR were further investigated in 100 patients (Table 2). No significant dose difference was observed for the esophagus, thyroid or humeral head between patients receiving IMN RT and those who did not. However, for the contralateral breast and contralateral lung, there was a significant (*p* < 0.001) increase in V_2Gy_ and V_4Gy_ between patient with and without IMN RT. Without IMN in the target volume, these parameters for the contralateral breast and the contralateral lung are zero, while inclusion of the IMN increases mean V_2Gy_ with approximately 10 cc for both structures.

**Table 2:** Dose-volume data for the esophagus, thyroid, humeral head, contralateral breast, and contralateral lung.

	IMN RT (n = 50) Median (IQR)	No IMN RT (n = 50) Median (IQR)	*p* *
D_mean, Esophagus_ [Gy]	1.2 (1.0 – 1.6)	1.0 (0.9 – 1.4)	0.2
D_mean, Thyroid_ [Gy]	11.9 (6.8 – 15.6)	13.0 (7.8 – 16.7)	0.4
D_mean, Humeral Head_ [Gy]	6.3 (4.3 – 9.9)	5.7 (3.3 – 9.1)	0.4
D_mean, PRV Humeral_ [Gy]	9.8 (7.0 – 12.2)	8.0 (6.1 – 11.2)	0.1
V_2Gy, ContraBreast_ [cc]	10.1 (2.6 – 27.6)	0 (0.0 – 0.0)	< 0.001
V_4Gy, ContraBreast_ [cc]	2.3 (0.1 – 13.9)	0 (0.0 – 0.0)	< 0.001
V_2Gy, ContraLung_ [cc]	11.3 (4.6 – 22.2)	0 (0.0 – 0.0)	< 0.001
V_4Gy, ContraLung_ [cc]	0.4 (0.0 – 3.3)	0 (0.0 – 0.0)	< 0.001

* *P*-value for an independent sample t-test (two-sided) was calculated for the contralateral breast and contralateral lung. For the esophagus, thyroid and humeral head a non-parametrical Mann–Whitney test was used.

cc: cubic centimeter; Gy: gray; IMN: internal mammary nodes; IQR: interquartile range; RT: radiotherapy.

### Irradiated and treated volume

Results for the IV and the TV are presented in Table 1. Both parameters showed a significant (*p* < 0.001) increase for patients receiving IMN RT compared to those without IMN RT. The TV was on average 2.6 times larger than the PTV volume for IMN RT patients. The corresponding number for patients without IMN RT, was a 2.1 times larger TV than PTV. The corresponding value for IV was 4.0 for IMN patients and 3.3 for patients without IMN RT.

### Target dose coverage

Both with and without IMN RT the clinical goal for PTV (breast or chest wall and regional lymph nodes) dose coverage was achieved. The mean D_98%_, along with 95% confidence intervals, was 93.0% (92.8% – 93.2%) without IMN RT and 93.4% (93.1% – 93.7%) with IMN RT.

**Figure 3:**
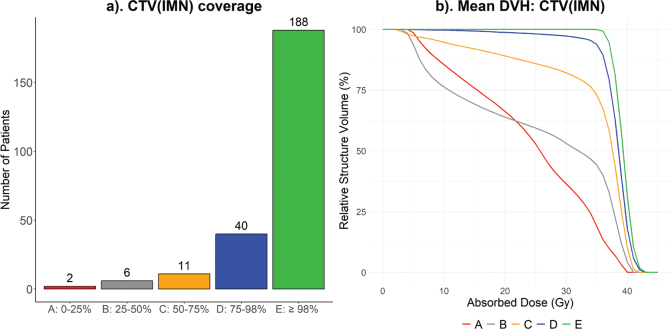
IMN-coverage categorized into five groups (A–E) depending on V_90%_ (a). Mean DVH for group A–E (b). CTV: clinical target volume; DVH: dose-volume histogram; IMN: internal mammary nodes.

Out of the 247 patients with IMN RT, 188 treatment plans successfully met the clinical goal for CTV_IMN_ coverage (V_90%_ ≥ 98%), while 59 treatment plans did not (V_90%_ < 98%). Coverage was categorized into five groups (A–E), depending on the minimum dose that 90% of the IMN structure volume received (i.e. V_90%_). This is presented in Figure 3.

### Incidence of pneumonitis

Clinical follow-up for at least 6 months after end of RT was available for 220 IMN patients. Of these, 2 (0.9%) were diagnosed with grade 2 pneumonitis, and no cases of grade 3 pneumonitis or higher were identified. The median follow-up time was 1.4-year, ranging from 0.5 to 4.0 years. In both cases of grade 2 pneumonitis, the BC was located in the right breast.

## Discussion

This study shows that most IMN patients (76%) were treated according to guidelines using standard 3D-CRT, without exceeding any dose constraints. When full CTV_IMN_ coverage could not be achieved, the primary limiting factor was the lung dose for all but one patient, consistent with findings from other studies [24]. The mean dose to the lung increased by approximately 2.7 Gy for IMN patients. Despite this, the incidence of symptomatic pneumonitis remained low.

The observed increase of 2.7 Gy in D_mean_ for the ipsilateral lung with the inclusion of IMN is in agreement with previous research [25]. The difference was more pronounced in patients treated on the left side. This may be explained by a smaller lung volume on the left side, the institutional routine of using DIBH for left-side treatments [26], and that for left sided treatments the field angle must be adjusted not only for the lung but also for the heart.

The absorbed dose to the lung during adjuvant RT for BC has been associated with an increased risk of developing a second primary lung cancer years after treatment. In a population-based study of Swedish women between 1992 and 2012, the cumulative incidence of lung cancer after 20 years follow-up was 3.0%, 2.3% and 2.0% for women with BC receiving RT, women with BC not receiving RT, and women without BC diagnoses, respectively [27].

A case-control study including more than 20,000 BC patients reported that the relative risk of lung cancer after BC RT increased linearly with whole lung mean dose at 8.5% per Gy, with an even higher excess relative risk of 17.3% per Gy for smokers [28]. In another study, an estimation of the absolute long-term risk to develop secondary lung cancer after modern BC RT was done in an individual patient data meta-analyses, including 75 trials and 40,781 women. The excess rate ratio (ERR) per Gy elevated mean dose to the whole lung was reported to be 0.11. Further they applied the ERR to population mortality data and estimated the absolute risk for secondary lung cancer from modern RT to be approximately 4% for long-term continuing smokers and 0.3% for non-smokers [3].

The given data cannot directly be correlated to the 2.7 Gy increase in mean lung dose seen in our study, since these studies used mean whole lung dose instead of mean dose to the ipsilateral lung, which is the variable available in our data. However, they clearly show the importance of keeping lung dose as low as reasonably possible.

Despite the increase in lung dose for patients receiving IMN RT, the incidence of pneumonitis was low. Few cases of grade 2 pneumonitis (moderate symptoms, medical intervention indicated and limited activities of daily living [23]) were reported, and no grade 3 (severe symptoms, limiting self-care activities of daily living and oxygen indicated [23]). This shows that also with the increased lung doses seen with IMN RT, symptomatic pneumonitis was rare. Since chest X-ray was not part of the routine follow-up, the overall incidence of grade 1 pneumonitis could not be evaluated.

These findings align with those of a phase III randomized trial that evaluated the incidence of pneumonitis during IMN RT. A total of 722 patients were randomly assigned to receive RT either with or without inclusion of the IMN in the target volume. Chest X-rays were performed prior to treatment and 6 months post-treatment. Pneumonitis grade 1 or 2 was reported in 4.8% of the patients, with no cases of grade 3 or higher observed. Notably, all grade 2 pneumonitis was observed in the IMN RT group, which also exhibited a higher overall rate of pneumonitis. Nevertheless, both the incidence and severity of pneumonitis were low and clinically acceptable [29].

For right-side and left-side treatments, IMN RT increased mean of D_mean_ to the heart with 0.3 Gy and 0.5 Gy, respectively. Studies have shown an association between BC RT and heart morbidity, which is correlated to the mean heart dose [3, 4]. However, the absolute risk increase caused by the treatment is strongly dependent on the patient’s individual health and initial risk of heart disease. Hence, for the general Swedish BC patient, the somewhat elevated heart dose with IMN RT should have limited clinical significance, although higher risks may be seen, for example, in patients who are smokers.

On the other hand, a difference in heart dose of 0.5 Gy is within the range of what can be achieved by using DIBH, and this has been widely adapted in order to reduce heart dose to BC patients worldwide [26, 30, 31]. It is not standard clinical practice to delineate the heart for right-sided treatments without IMN. As a result, the heart structure was missing for part of this patient group in our study.

No significant dose differences were observed for the esophagus, thyroid, or the humeral head. This was due to the anteroposterior field assessment in this area. Including IMN in the target did not affect the field arrangement and therefore did not change dose to these OAR. On the other hand, there was a significant difference in V_2Gy_ and V_4Gy_ for both the contralateral lung and contralateral breast. There were also significant differences in IV and TV. These results can be explained by a difference in field angle when including IMN in the RT field, which enlarges the overall irradiated volume.

The increase in IV and TV confirms that including the IMN in the target not only expands the volume receiving a high dose but also increases the intermediate dose levels to surrounding tissue. The IV and TV relative to PTV volume increased by approximately 21% and 23%, respectively, while treating the IMN. To improve conformity of the TV, other treatment planning techniques have been utilized, for example volumetric modulated arc therapy (VMAT) [32, 33], intensity-modulated RT [34], or proton therapy [35]. While these techniques offer advantages in conformity of the high-dose region, they also come with altered distribution of the low-dose bath, potentially increasing exposure to OARs that would receive minimal or no dose using 3D-CRT [36]. The robustness of VMAT to anatomical changes (e.g. breast swelling) has been shown to be comparable to that of the 3D-CRT technique, which inherently accounts for such variations [32, 33].

The desired CTV_IMN_ dose coverage (V_90%_ ≥ 98%) was met for 76% of the patients, and the limiting factor for successful coverage was the lung dose. Upon closer examination of the group receiving V_90%_ between 75%-98% (group D), 24 of the 40 patients fulfilled V_90%_ ≥ 90% and 13 of these patients also fulfilled V_90%_ ≥ 95%. The loss of coverage in this group was primarily behind the supraclavicular joint, which is acceptable according to ESTRO guidelines, and requires no intervention during treatment planning [21]. This means that the desired CTV_IMN_ coverage was obtained, or very close to obtained, for 86% of treatment plans. Which is a quite high target coverage compared to other studies on IMN RT [18].

When discussing IMN coverage, previous investigations have shown that the IMN is exposed to incidental dose during breast or chest wall irradiation due to its proximity to the target volume. In one study, 73% of patients undergoing breast or chest wall irradiation received complete or partial incidental dose to the IMN. These results raise the question of whether the incidental dose has a therapeutic effect and, together with modern systemic therapy, helps prevent recurrence in the IMN [37, 38].

Our study has some limitations. Unfortunately, we did not have data on frequency of pneumonitis for patients without IMN RT. Neither was the heart delineated for all patients with right-sided tumors. Some patients living outside the region were lost to follow-up, and not all treatment plans could be reviewed. Still, the study includes a large number of patients, showing how IMN RT affects dose to OAR and target coverage in the clinical setting. Although the data are from Swedish patients, the cohort includes patients with anatomical variations, hence the findings should be generalizable to other BC populations.

Indications for IMN RT and the prioritization of target coverage versus dose to OAR differ between countries and sites. On the one hand, IMN RT has been shown to be beneficial in high-risk patients, on the other hand treatment comes with an increased absorbed dose to the heart and lungs. Hence, there is an ongoing debate on the risk-benefit with this treatment, especially in patients with a low/intermediate risk of regional recurrence. Previous studies investigating the absolute increase in dose to OAR with IMN RT in the clinical setting are sparse. Hence, we believe this study provides important information for further discussions on BC patients who should receive IMN RT.

In conclusion, we found the introduction of IMN RT in the clinic to primarily result in an increased lung dose. Still, the rate of symptomatic pneumonitis was low. IMN RT gave a significantly higher heart dose; however, the absolute increase in gray was relatively small. Additionally, IMN RT increased the IV and the TV. For most patients, the desired IMN coverage could be achieved using traditional 3D-CRT technique. In cases where dose coverage could not be attained the lung dose was the limiting factor.

## Data Availability

The data that support the findings of this study are available from the authors upon reasonable request.
